# Mortality trends of Oral Cancer in the United States: a retrospective analysis

**DOI:** 10.3389/fonc.2026.1850969

**Published:** 2026-06-30

**Authors:** Xiao Li, Qian Zhou, Huanhuan Li

**Affiliations:** 1 Zhejiang University School of Medicine Second Affiliated Hospital Linping Hospital, Department of Stomatology, Hangzhou, Zhejiang, China; 2 School of Nursing, Anhui Medical University, Hefei, Anhui, China

**Keywords:** age-adjusted mortality rate, CDC WONDER, disparities, mortality trends, oral cancer, United States

## Abstract

**Background:**

Oral cancer constitutes a substantial global public health challenge with considerable mortality and socioeconomic burden. This study aimed to examine temporal trends in oral cancer mortality from 1999 to 2024.

**Methods:**

Population-level mortality data were derived from the CDC WONDER online database (https://wonder.cdc.gov/mcd-icd10.html), with raw data formally extracted on January 12, 2026. Age-adjusted mortality rates (AAMRs) were calculated for each stratification. Joinpoint regression was used to estimate annual percentage change (APC) and average annual percentage change (AAPC) to identify statistically significant trends.

**Results:**

From 1999 to 2024, total oral cancer deaths in the United States rose from 7,451 to 12,368, corresponding to a 65.99% increase, whereas the overall AAMR remained stable (P > 0.05). Females showed a significant decrease in AAMR (P < 0.05), while males exhibited no significant change. By census region, the Midwest showed a significant increasing trend (P < 0.05), and the West displayed a significant decreasing trend (P < 0.05). Non-Hispanic Black individuals experienced the sharpest reduction (P < 0.05), whereas non-Hispanic White individuals showed a significant increase (P < 0.05). Metropolitan and nonmetropolitan mortality trend analyses were confined to 1999–2020; within this restricted timeframe, metropolitan areas had a significant declining AAMR (P < 0.05), while nonmetropolitan areas increased significantly (P < 0.05). Mortality rates decreased significantly among adults aged 35–54 years but increased markedly among those aged 65 years and older, especially individuals aged 85 years and older.

**Conclusion:**

Although total oral cancer deaths increased substantially between 1999 and 2024, overall age-adjusted mortality remained unchanged. Pronounced and persistent sociodemographic disparities exist across sex, region, race/ethnicity, urban-rural status, and age. Observed divergent mortality patterns represent descriptive population-level disparities, and causal links to healthcare access, behavioural risks or clinical treatment cannot be definitively inferred from current ecological mortality data alone.

## Introduction

Oral cancer, encompassing malignancies of the lip, tongue, gingiva, floor of the mouth, and other oral cavity sites, represents a leading cause of cancer-related mortality worldwide (1, 2). In the United States, oral cancer is associated with well-established risk factors including tobacco use, alcohol consumption, human papillomavirus (HPV) infection, and low socioeconomic status (3, 4). Despite advances in early detection strategies, surgical interventions, and multimodal therapy, long-term survival for advanced-stage oral cancer remains suboptimal, and mortality disparities across demographic subgroups persist (5, 6).

Long-term population-level surveillance of cancer mortality is critical for evaluating the effectiveness of public health policies, allocating healthcare resources, and identifying vulnerable populations (7). The CDC WONDER database provides comprehensive, nationally representative mortality data that enable robust assessment of temporal trends and sociodemographic variations (8).While prior studies have examined oral cancer incidence or survival, few have provided a comprehensive, multi-stratified analysis of mortality trends spanning 1999–2024, including sex, census region, race/ethnicity, urbanization, and age.

Using the CDC WONDER database, this retrospective population-based study aimed to: (1) characterize overall trends in oral cancer mortality in the United States from 1999 to 2024; (2) evaluate mortality disparities by sex, census region, race/ethnicity, urbanization level, and age group; and (3) identify statistically significant temporal changes using Joinpoint regression. The findings are intended to inform evidence-based interventions to reduce oral cancer mortality and mitigate health inequities.

## Methods

### Data source

Mortality data for oral cancer were obtained from the Centers for Disease Control and Prevention WONDER underlying Cause of Death ICD-10 database (MCD-ICD10, official access URL: https://wonder.cdc.gov/mcd-icd10.html) covering calendar years 1999 to 2024; urban–rural stratified mortality data are only publicly available from CDC WONDER for 1999–2020, hence urban-rural analyses were restricted to this shorter period. Raw aggregated dataset was downloaded and locally archived on January 12, 2026, with fixed standardized query parameters pre-set before database extraction. Oral cancer was strictly defined using full International Classification of Diseases, 10th Revision (ICD-10) codes C00–C14, covering malignant neoplasm of lip (C00), base of tongue (C01), other unspecified parts of tongue (C02), gum (C03), floor of mouth (C04), palate (C05), other and unspecified parts of mouth (C06), tonsil (C09), oropharynx (C10), nasopharynx (C11), pyriform sinus (C12), hypopharynx (C13), other ill-defined sites within lip, oral cavity and pharynx (C14). Records were exclusively included if oral cancer (C00–C14) was coded as the underlying cause of death per death certificate coding rules defined by CDC National Center for Health Statistics (NCHS); multiple-cause-of-death entries without oral cancer listed as underlying cause were excluded from the analysis. This study was exempt from institutional review board approval because the CDC WONDER database contains only de-identified population-level data and complies with the Strengthening the Reporting of Observational Studies in Epidemiology (STROBE) guidelines.

### Data extraction

Data were extracted for all U.S. states and the District of Columbia following unified CDC WONDER query rules: CDC default suppression thresholds were strictly followed: all cells with fewer than 10 death counts were automatically excluded per CDC data reliability standards to avoid unstable/suppressed statistical estimates; no imputation or replacement was performed for suppressed values. Population denominators used for mortality rate calculation were retrieved from embedded NCHS bridged-race intercensal and postcensal US population estimates preloaded within the MCD-ICD10 CDC WONDER platform. All final raw exported CSV datasets and complete Joinpoint command/analysis code files are uploaded as **Supplementary Material File S1** and **S2** for independent replication. Cases were stratified by sex, census region, race/ethnicity, urbanization level (only 1999–2020), and age group. Sex was categorized as male or female. Census regions were classified as Northeast, Midwest, South, and West as defined by the U.S. Census Bureau official four-region grouping standard. Race/ethnicity was categorized into four mutually exclusive categories following CDC NCHS bridged race specification: Hispanic (all origin races, any ethnicity), non-Hispanic Black (single Black race, non-Hispanic ethnicity), non-Hispanic White (single White race, non-Hispanic ethnicity), non-Hispanic Other (aggregated Asian, Native Hawaiian, Pacific Islander, multiracial individuals with non-Hispanic background per CDC grouping definitions). Urbanization level was classified as metropolitan or nonmetropolitan according to the 2013 National Center for Health Statistics (NCHS) Urban-Rural Classification Scheme (9), with county-level urban/rural mapping fixed per NCHS 2013 standard, and this stratification variable unavailable for 2021–2024 in CDC WONDER. Age groups were defined as 25–34, 35–44, 45–54, 55–64, 65–74, 75–84, and 85 years and older.

### Statistical analysis

Age-adjusted mortality rates (AAMRs) standardized to the 2000 U.S. standard population were calculated for sex, census region, race/ethnicity, and urbanization level. Crude mortality rates (CMRs) were used for age-group analyses. The Joinpoint Regression Program (version 5.0, National Cancer Institute) was used to estimate annual percent change (APC) and average annual percent change (AAPC) with 95% confidence intervals (CIs). Two-tailed tests were performed, and P < 0.05 was considered statistically significant.

## Results

### Overall mortality trends (1999–2024)

From 1999 to 2024, the total number of oral cancer–related deaths in the United States increased from 7451 to 12368, representing a 65.99% change. In contrast, the age-adjusted mortality rate (AAMR) remained relatively stable over the same period, from 4.20 per 100,000 population (95% CI: 4.10 to 4.30) in 1999 to 4.28 per 100,000 (95% CI: 4.21 to 4.36) in 2024, with an average annual percent change (AAPC) of −0.06 (95% CI: −0.32 to 0.20, P > 0.05) (**Table 1**).

**Table 1:** *Oral Cancer* causes deaths and AAMR in the United States from 1999 to 2024 and their changing trends.

Characteristic	Deaths			AAMR		
	1999	2024	Percent change (%)	1999 (95% CI)	2024 (95% CI)	AAPC (95% CI)
Total	7451	12368	65.99	4.20 (4.10 to 4.30)	4.28 (4.21 to 4.36)	-0.06 (-0.32 to 0.20)
Sex						
Female	2531	3559	40.62	2.48 (2.38 to 2.57)	2.30 (2.22 to 2.38)	-0.28 (-0.54 to -0.01)*
Male	4920	8809	79.04	6.38 (6.20 to 6.56)	6.55 (6.41 to 6.69)	0.02 (-0.25 to 0.29)
Census Region						
Northeast	1448	1946	34.39	3.97 (3.76 to 4.17)	3.66 (3.49 to 3.83)	-0.29 (-0.66 to 0.09)
Midwest	1583	2798	76.75	3.79 (3.60 to 3.98)	4.61 (4.44 to 4.79)	0.62 (0.31 to 0.93)*
South	2946	5030	70.74	4.69 (4.52 to 4.86)	4.52 (4.40 to 4.65)	-0.08 (-0.56 to 0.40)
West	1474	2594	75.98	4.10 (3.89 to 4.31)	3.92 (3.77 to 4.07)	-0.29 (-0.58 to 0.00)*
Race						
Hispanic	281	778	176.87	2.88 (2.53 to 3.24)	2.34 (2.17 to 2.52)	-0.90 (-1.60 to -0.21)*
NH Black	1121	1100	-1.87	6.79 (6.38 to 7.19)	3.64 (3.43 to 3.87)	-2.46 (-2.90 to -2.01)*
NH White	5807	9826	69.21	3.99 (3.89 to 4.09)	4.80 (4.70 to 4.89)	0.70 (0.38 to 1.03)*
NH Other	219	1268	479.00	3.57 (3.07 to 4.07)	3.53 (3.33 to 3.73)	-0.13 (-0.99 to 0.74)
Urbanization^1^						
Metropolitan	6107	9773	60.01	4.22 (4.12 to 4.33)	3.80 (3.72 to 3.88)	-0.54 (-0.79 to -0.29)*
Nonmetropolitan	1344	2595	93.08	4.15 (3.93 to 4.38)	4.74 (4.53 to 4.96)	0.53 (0.30 to 0.75)*
Age Groups^2^						
25-34 years	43	73	69.77	0.11 (0.08 to 0.14)	0.16 (0.12 to 0.20)	0.18 (-0.54 to 0.90)
35-44 years	291	207	-28.87	0.65 (0.57 to 0.72)	0.45 (0.39 to 0.52)	-1.66 (-2.02 to -1.30)*
45-54 years	1061	800	-24.60	2.90 (2.73 to 3.08)	1.96 (1.83 to 2.10)	-1.43 (-1.66 to -1.19)*
55-64 years	1616	2766	71.16	6.80 (6.46 to 7.13)	6.64 (6.39 to 6.89)	-0.24 (-0.66 to 0.19)
65-74 years	1896	3896	105.49	10.29 (9.83 to 10.76)	10.99 (10.65 to 11.34)	0.13 (-0.05 to 0.31)
75-84 years	1744	3082	76.72	14.27 (13.60 to 14.94)	15.97 (15.41 to 16.53)	0.77 (0.38 to 1.17)*
85+ years	800	1544	93.00	19.26 (17.92 to 20.59)	23.99 (22.80 to 25.19)	0.85 (0.37 to 1.33)*

*P<0.05· ^1^In the context of urbanization, the 2024 AAMR data was substituted with that from 2020, and the AAPC was calculated based on the period from 1999 to 2020· ^2^For the age groups, the crude mortality rate was used as a substitute for AAMR, and the AAPC was computed based on the crude mortality rate. AAMR, age-adjusted mortality rate; CI, confidence interval; AAPC, average annual percent change; NH, non-Hispanic.

### Mortality by sex

**Figure 1:**
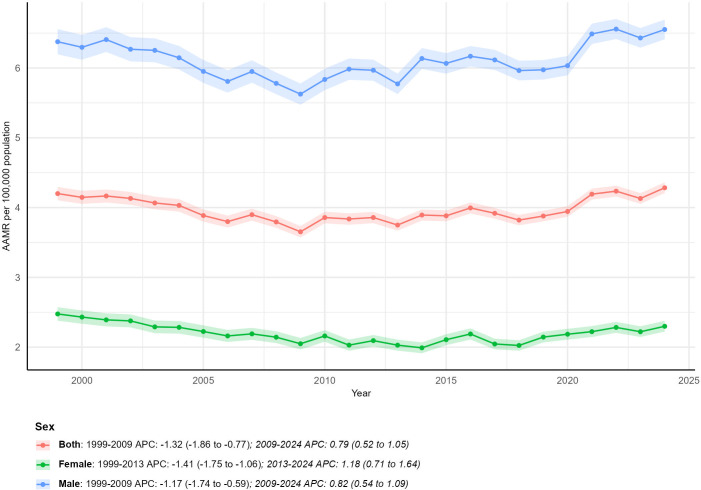
Sex-stratified age-adjusted oral cancer mortality rates in the U.S., 1999–2024.

Sex-specific differences in mortality were sustained throughout the study period. Female deaths increased from 2531 to 3559, a 40.62% change, while male deaths rose from 4920 to 8809, a 79.04% change. Males consistently exhibited higher AAMRs than females. The AAMR for females decreased significantly from 2.48 (95% CI: 2.38 to 2.57) to 2.30 (95% CI: 2.22 to 2.38), with an AAPC of −0.28 (95% CI: −0.54 to −0.01, P < 0.05). In comparison, the AAMR for males showed no statistically significant change, with an AAPC of 0.02 (95% CI: −0.25 to 0.29, P > 0.05) (**Figure 1**).

### Mortality by census region

**Figure 2:**
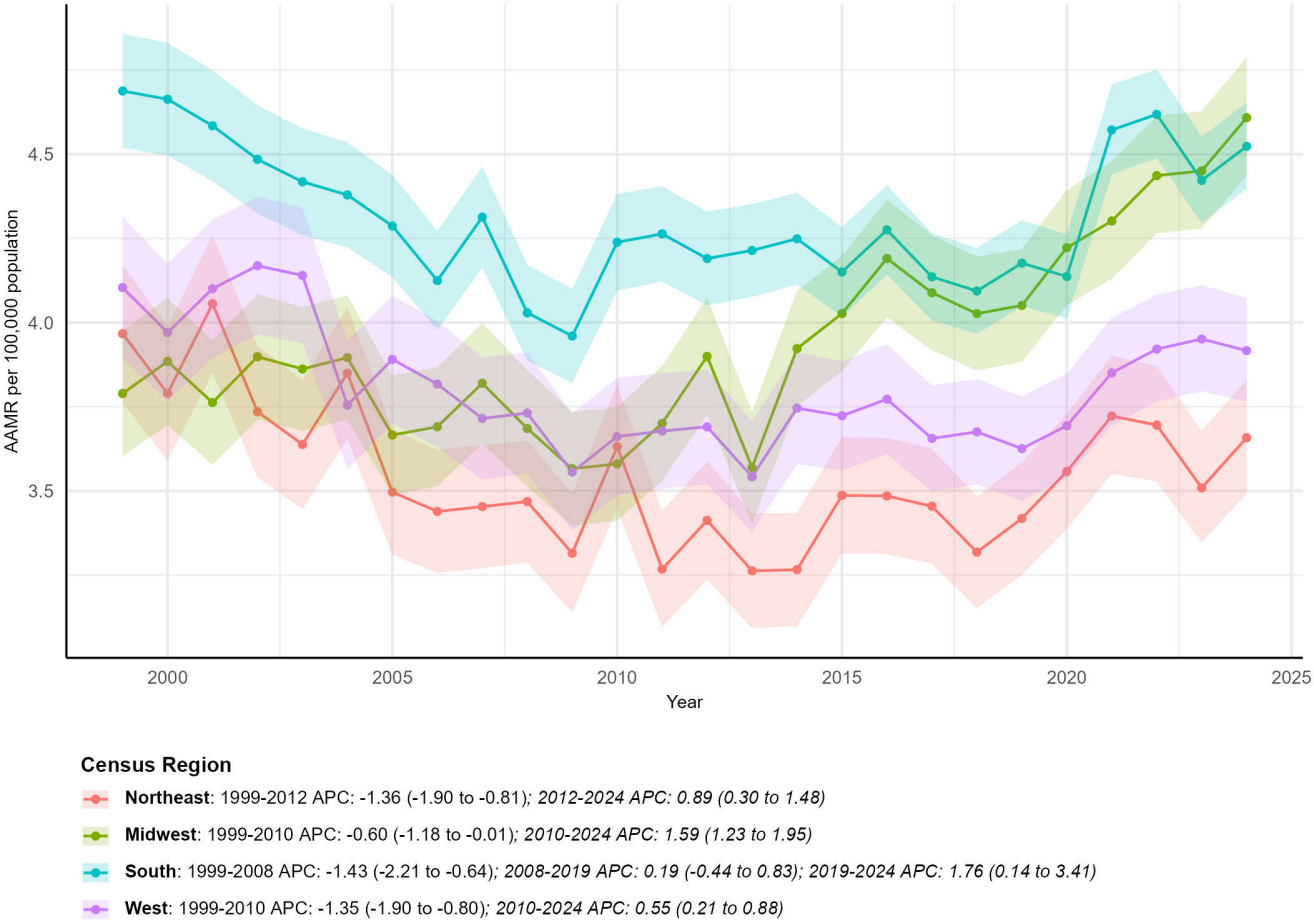
Regional age-adjusted oral cancer mortality rates across four U.S. census regions, 1999–2024.

Distinct regional disparities were observed in oral cancer mortality trends. The Northeast region recorded a 34.39% increase in deaths, with a non-significant downward AAMR trend. The Midwest showed a 76.75% increase in deaths and a significant upward trend in AAMR, with an AAPC of 0.62 (95% CI: 0.31 to 0.93, P < 0.05). The South region had a 70.74% increase in deaths with a stable AAMR trend. The West region showed a 75.98% increase in deaths and a significant decreasing AAMR trend, with an AAPC of −0.29 (95% CI: −0.58 to 0.00, P < 0.05) (**Figure 2**).

### Mortality by race/ethnicity

**Figure 3:**
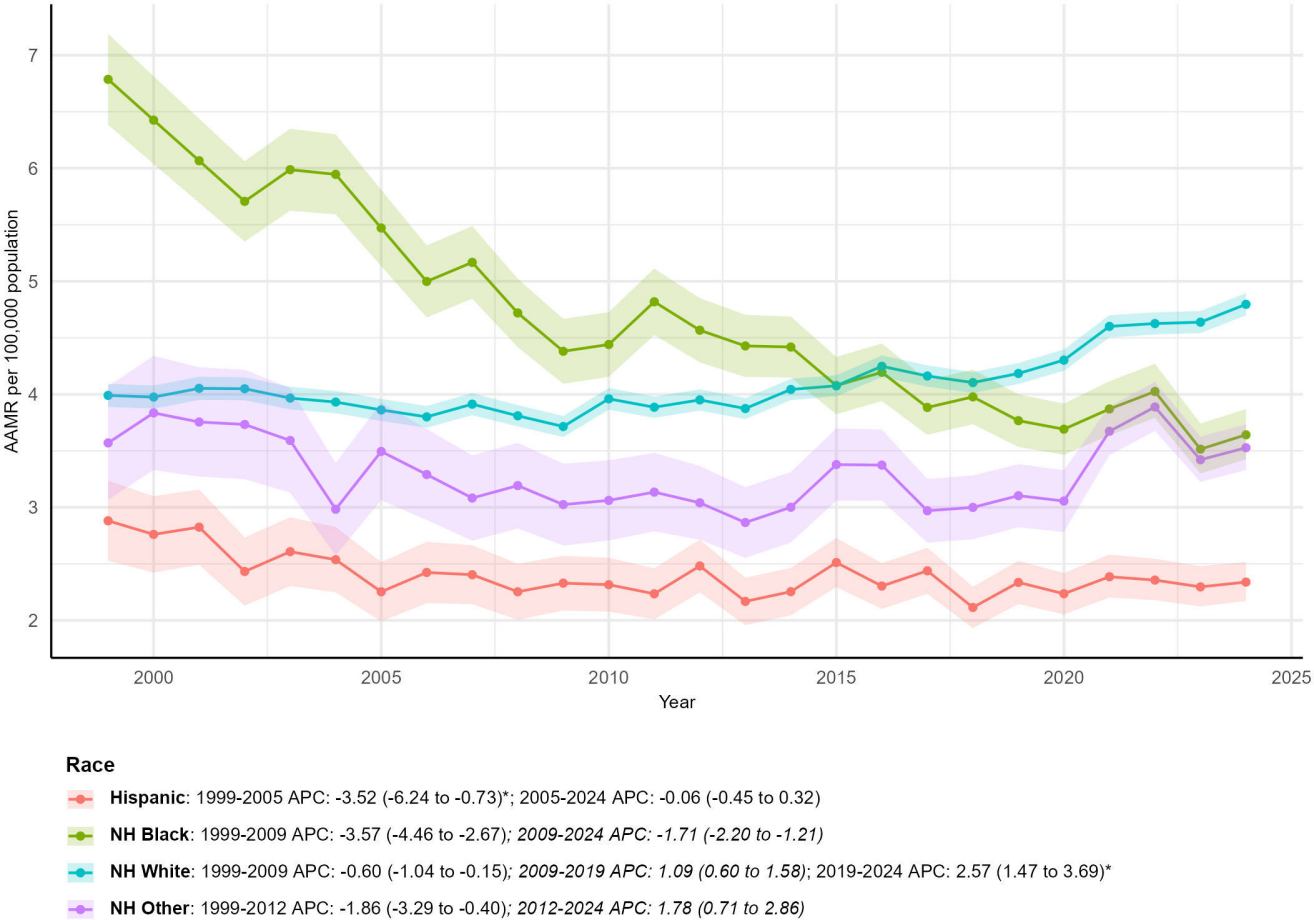
Race/ethnicity-stratified age-adjusted oral cancer mortality rates, U.S. 1999–2024.

Marked racial and ethnic disparities were identified in oral cancer mortality. Hispanic individuals experienced a 176.87% increase in deaths, accompanied by a significant reduction in AAMR (AAPC = −0.90, 95% CI: −1.60 to −0.21, P < 0.05). Non-Hispanic Black individuals showed a slight 1.87% decrease in deaths and the most pronounced decline in AAMR, with an AAPC of −2.46 (95% CI: −2.90 to −2.01, P < 0.05). Non-Hispanic White individuals had a 69.21% increase in deaths and a significant increasing AAMR trend (AAPC = 0.70, 95% CI: 0.38 to 1.03, P < 0.05). Non-Hispanic Other individuals showed the largest relative increase in deaths (479.00%) but no significant change in AAMR (**Figure 3**).

### Mortality by urbanization level

**Figure 4:**
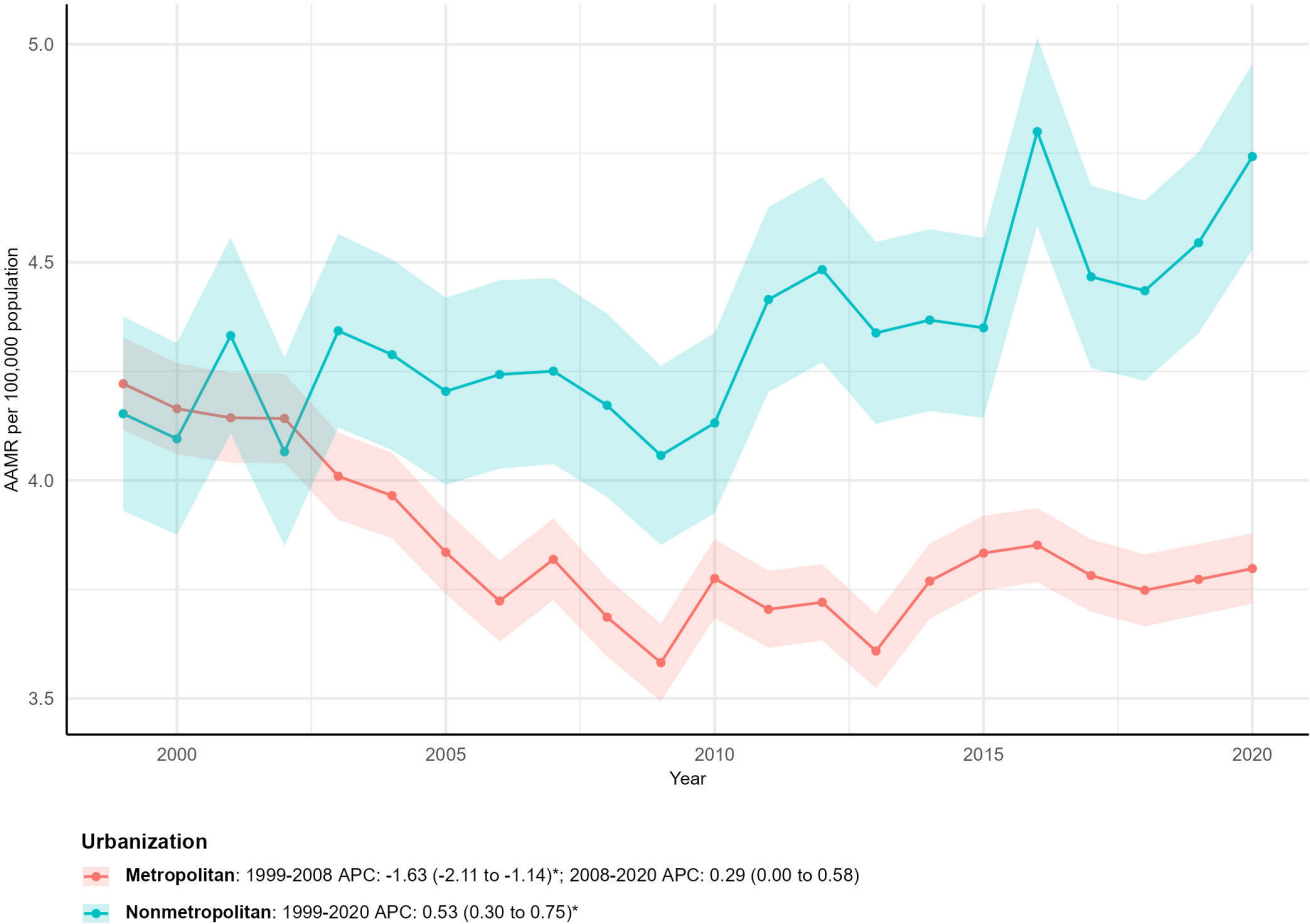
Urban-rural age-adjusted oral cancer mortality rates , U.S. 1999–2020.

Urban–rural stratified mortality analysis was limited to calendar years 1999–2020 due to unavailable NCHS urban-rural classification data in CDC WONDER for years 2021–2024; all subsequent results in this subsection only apply to 1999–2020. Within this restricted period, metropolitan areas reported a 60.01% increase in deaths and a significant decreasing AAMR trend, with an AAPC of −0.54 (95% CI: −0.79 to −0.29, P < 0.05). Nonmetropolitan areas showed a 93.08% increase in deaths and a significant increasing AAMR trend (AAPC = 0.53, 95% CI: 0.30 to 0.75, P < 0.05). Nonmetropolitan areas consistently maintained higher AAMRs than metropolitan areas across the 1999–2020 study window (**Figure 4**).

### Mortality by age group

**Figure 5:**
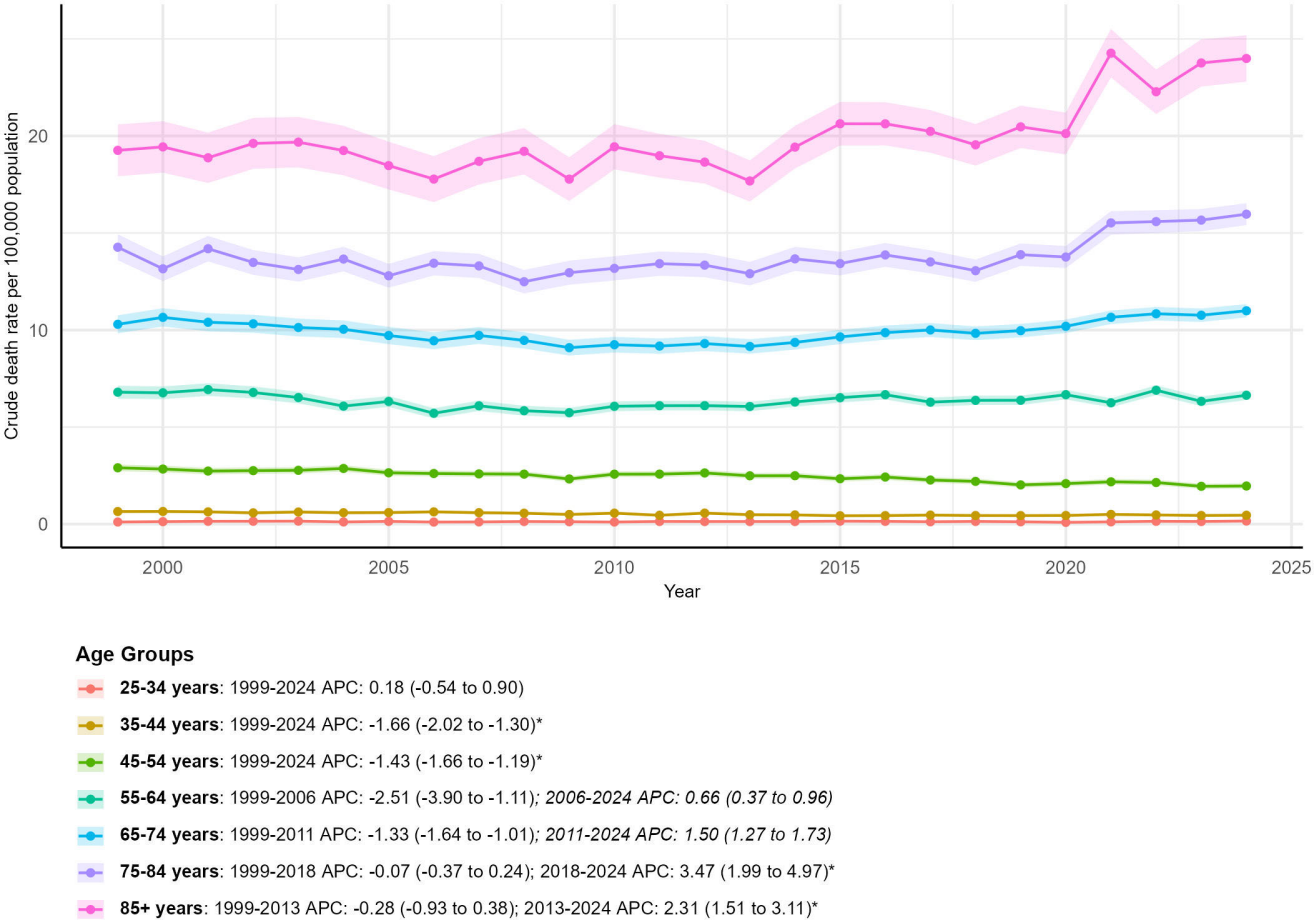
Age-group crude oral cancer mortality rates in the U.S., 1999–2024.

Crude mortality rates varied widely across age groups and demonstrated divergent trends. Individuals aged 25–34 years showed a 69.77% increase in deaths with no significant trend. Those aged 35–44 years and 45–54 years showed significant decreases in mortality, with AAPCs of −1.66 (P < 0.05) and −1.43 (P < 0.05), respectively. The 55–64 and 65–74 age groups showed no significant trends. In contrast, individuals aged 75–84 years and 85 years and older had substantial increases in deaths and significant upward mortality trends, with AAPCs of 0.77 (P < 0.05) and 0.85 (P < 0.05), respectively. The oldest age group (85 years and older) carried the highest crude mortality rate throughout the study period (**Figure 5**).

## Discussion

This nationwide analysis of oral cancer mortality in the United States from 1999 to 2024 yields three principal findings. First, absolute deaths increased substantially, likely driven by population growth and aging, while overall age-adjusted mortality remained unchanged. Second, pronounced disparities persisted across all sociodemographic strata, with divergent trends by sex, region, race/ethnicity, urban-rural status, and age. Third, middle-aged adults experienced mortality reductions, whereas older adults bore an increasing burden, particularly those aged 85 years and older.

The stable overall AAMR despite rising absolute deaths may reflect the competing effects of population aging and improvements in prevention, diagnosis, and treatment (10, 11). Observed declining mortality among females and younger middle-aged adults represents an epidemiologic association; we cannot confirm causal contributions from reduced tobacco use or improved public awareness based solely on current ecological mortality data (12). In contrast, the increasing burden among males, the Midwest, nonmetropolitan regions, and NH White individuals is documented as a descriptive population disparity, and definitive causal connections to uneven access to prevention, screening, and specialized care cannot be established from the present mortality dataset alone (13, 14).

Racial and ethnic disparities were striking. NH Black individuals experienced the most rapid mortality decline; this population-level trend is reported descriptively, and causal links to targeted public health campaigns or modified traditional risk factor exposure are speculative and unproven with available study data (15, 16). Meanwhile, NH White individuals showed a significant increase, which may correlate with high-risk behaviours, delayed diagnosis or differential care access, yet causal inference is beyond the scope of this population-based mortality analysis. Hispanic and NH Other populations exhibited large increases in absolute deaths, likely correlated with demographic growth and structural healthcare barriers, though formal causal verification is not feasible herein (17).

Urbanrural disparities were pronounced (analyzed 1999–2020 only). Nonmetropolitan areas showed rising mortality rates, consistent with previously reported epidemiologic correlations of limited local medical workforce and higher risky substance use in rural cohorts; direct causation cannot be concluded from our mortality trend data alone (18, 19). Metropolitan areas benefited from concentrated healthcare resources and earlier adoption of screening and treatment advances per existing literature associations.

Agerelated patterns highlight the critical impact of the aging U.S. population. Mortality rates decreased among younger and middleaged adults, likely due to risk reduction and early detection based on published correlative research. In contrast, adults aged 65 years and older experienced substantial increases, attributable to greater frailty, comorbidities, and less aggressive screening and treatment in older populations as previously documented; all proposed contributing factors remain correlative rather than causally confirmed by our mortality dataset (20, 21).

However, several limitations must be acknowledged. First, Urban–rural analyses are limited to 1999–2020 due to incomplete publicly available CDC WONDER urban-rural classification data for years after 2020. Second, the database lacks clinical details such as tumor stage, histology, and treatment information, which prevents analysis of mortality trends by disease severity or therapeutic approach.Finally, potential misclassification of cause of death, such as attributing death to oral cancer, which may introduce bias, particularly in older adults with multiple chronic conditions.

## Conclusion

Oral cancer mortality in the United States from 1999 to 2024 was characterized by rising absolute deaths, stable overall ageadjusted mortality, and profound sociodemographic disparities; urban-rural disparity assessment was restricted to 1999–2020 per source data constraints. Males, the Midwest, nonmetropolitan areas (1999–2020), NH White individuals, and older adults carry an increasingly disproportionate burden. These results support targeted interventions including: expanded access to screening in rural and underserved regions; culturally appropriate education; HPV vaccination promotion; and strengthened geriatric oncology care. Sustained investment in equitable prevention and treatment is essential to reduce oral cancer mortality and eliminate disparities. Notably, all identified disparities are descriptive statistical associations; the present study cannot prove causal relationships between healthcare service, behavioural factors and observed mortality divergence.

## Data Availability

The original contributions presented in the study are included in the article/**Supplementary Material**. Further inquiries can be directed to the corresponding author.
